# Job Demands, Resources, and Burnout in Social Workers in China: Mediation Effect of Mindfulness

**DOI:** 10.3390/ijerph181910526

**Published:** 2021-10-07

**Authors:** Chienchung Huang, Xiaoxia Xie, Shannon P. Cheung, Yuqing Zhou, Ganghui Ying

**Affiliations:** 1 School of Social Work, Rutgers University, New Brunswick, NJ 08901, USA; huangc@ssw.rutgers.edu (C.H.); scheung@ssw.rutgers.edu (S.P.C.); 2 Research Institute of Social Development, Southwestern University of Finance & Economics, Chengdu 611130, China; zhouyuqing@smail.swufe.edu.cn (Y.Z.); ygh@smail.swufe.edu.cn (G.Y.)

**Keywords:** job demands, resources, mindfulness, burnout, social workers, China

## Abstract

Internationally, human service professionals, including social workers, experience high burnout and turnover rates. Despite the recent and rapid development of contemporary social work in China, Chinese social workers similarly experience significant rates of burnout. Therefore, there is a need to investigate the factors that contribute to social work burnout. This study applied the job demands and resources (JD-R) model to examine the effects of JD-R on burnout in social workers (*n* = 897) from Chengdu, China, and whether these relations are mediated by state mindfulness. Structural equation modeling results supported the previously hypothesized dual process by which JD-R affect burnout, specifically in a sample of social workers in China. Job demands (JD) were positively associated with burnout, while job resources (JR) were negatively associated with burnout. These relations were partially mediated by state mindfulness. JR had a strong, positive direct effect on mindfulness (β = 0.38), and its total effect on burnout was high (β = −0.56). Meanwhile, JD had a slight negative direct effect on mindfulness (β = −0.09), and its total effect on burnout was 0.42. The results suggest that the implementation of mindfulness-based interventions for social workers can potentially mitigate the effect of JD on burnout, as well as increase the effect of JR on burnout.

## 1. Introduction

Recent decades have seen an increase in the scholarly literature on stress, burnout, and turnover in the helping professions. Empirical research has suggested that social workers may experience higher levels of stress and, subsequently, burnout than comparable occupational groups [1,2]. Indeed, despite its recent and rapid development of contemporary social work, China has also experienced high turnover and burnout rates among its social workers [3,4,5,6]. This raises significant concerns over the future of social work in China, as burnout can jeopardize social workers’ physical health [7] and mental health [8] and is positively associated with turnover [9,10,11,12]. High burnout and turnover have negative implications for job performance [13] and, therefore, the quality of services [14,15]. This is significant, given that in China, social workers provide services to vulnerable communities in settings such as schools, hospitals, government agencies, and community centers [16,17]. Despite this, little scholarly research has examined the factors underlying burnout among Chinese social workers. This study thus endeavors to better understand job-related factors that contribute to and protect against burnout in a sample of Chinese social workers from Chengdu, China. We also examine how state mindfulness might serve as a mediator in such relations. The results of this study can be applied to better support social workers’ occupational well-being and to inform interventions that seek to mitigate the risk of burnout among Chinese social workers.

## 2. Literature Review, Theory, and Conceptual Framework

### 2.1. The Current State of Social Work in China

Since 1979, China has experienced rapid economic development. This, however, has come with increased social issues and problems, including rural poverty, labor migration, and a growing population of migrant and left-behind children [18,19,20,21,22]. In response to these developing social problems, the social work profession in China has substantially expanded, as evidenced by the growth of formal social work degree programs. Peking University began recruiting undergraduate social work students in 1989. By the end of 1999, China had launched 27 social work undergraduate programs. The rate at which social work programs were established grew faster after 2000, with 20 to 30 programs established each year [23]. By the end of 2018, there were 348 social work undergraduate programs, 147 Master of Social Work (MSW) programs, and 17 Ph.D. programs in China, together producing over 40,000 social work graduates each year [16]. While these programs lack clear standardization guidelines, the professionalization of social work has accelerated tremendously [23]. In fact, the profession grew from 0.2 million social workers in 2010 to 1.2 million in 2018 [16,23]. At the same time, scholars have observed considerable turnover among social workers; 25% of social workers in Guangzhou, the capital city of Guangdong province, had left their jobs in 2014 [6]. Meanwhile, in Shenzhen, the city that links Hong Kong to mainland China, the turnover rate of social workers grew from 8.2% to 18.08% between 2008 and 2015 [3]. Although statistics regarding social work turnover from other cities are scarce, local government officers in Beijing estimated that 25% of social workers had left their jobs in 2014 [24], and this percentage was even higher in Dong Guan, where over 50% of social workers had considered quitting in 2014 [25].

### 2.2. Job Burnout

Given that burnout is strongly and positively associated with turnover [9,10,11,26,27], it is prudent to investigate the factors that precede burnout among social workers in China. Burnout, a multidimensional construct that encompasses exhaustion, cynicism, and sense of inefficacy [28], has long been recognized as an occupational hazard for human service professionals [13,29,30,31], social workers included [32,33]. Burnout has been found to positively predict job absenteeism [32,34,35] and poor work performance [13]. In addition, burnout poses health risks—both physical [35,36] and mental [37,38]—to working professionals.

### 2.3. The JD-R Theory and Burnout

The job demands and resources (JD-R) theory has been proposed to explain how working conditions (i.e., job demands [JD] and job resources [JR]) affect individuals’ experiences of job burnout [39,40,41,42]. The JD-R theory posits that high JD, through a health impairment process, can deplete individuals’ energy and lead to burnout. JD are defined as the physical, social, or organizational aspects of a job that require sustained physical or mental effort. Meeting different JD comes at psychological costs (e.g., exhaustion). By contrast, JR are aspects of the job (e.g., physical, psychological, social, or organizational) that may support individuals in achieving their work goals. JR may serve to reduce burnout through a motivational process [40], in which they reduce the associated psychological costs of JD or stimulate individuals’ personal growth [41]. Without JR, individuals may face challenges barring them from completing tasks efficiently, leading to frustration, withdrawal, and disengagement. Since its original conceptualization, the JD-R model has been applied in a multitude of cross-cultural studies to examine the occupational well-being of professionals across several disciplines and industries [35,39,41,43,44,45,46,47]. Most notably, one study analyzed data collected from approximately 750 social workers in Guangzhou, China, and found that JD significantly increased burnout and intention to leave their jobs, while JR reduced burnout and turnover [26]. Given that the JD-R model emphasizes environmental conditions on the job, few studies have examined whether internal experiences, such as state mindfulness, affect the relation between JD-R and burnout. Past studies have shown how mindfulness can act as a buffer against the negative effects of life stressors [48,49,50,51]. In fact, in one study of 79 urban firefighters, mindfulness was associated with positive affect on days that participants reported greater work stress [51]. It follows, then, that mindfulness may affect the relation between JD-R and burnout in social workers, who similarly face a great deal of stressors on the job.

### 2.4. Mindfulness

Mindfulness describes a state of consciousness during which an individual actively engages in purposeful awareness and attention to the present moment, while maintaining non-judgmental reactions to their observations [52,53]. Two key components of mindfulness, a multi-dimensional construct, are mindful attention and mindful metacognition. The former is known to regulate individuals’ attention by focusing on awareness of the present moment. On the other hand, mindful metacognition, also known as decentering, is the detachment of oneself from monitoring of thoughts and feelings about ongoing events. To remain non-judgmental or neutral about the present moment, individuals may acknowledge—or “let go of”—the feelings and thoughts that arise in response to the people and events that they observe [54,55]. Mindfulness has traitlike properties in that it varies among individuals and from moment to moment [54,56].

Studies on mindfulness and its association with a myriad of positive effects have proliferated in recent decades. In these studies, mindfulness is found to be associated with positive outcomes including social and emotional competence [57,58], as well as well-being and perceived health [59]. At the same time, however, studies have shown that negative life experiences can reduce an individual’s mindfulness [60,61,62,63]. In addition, past research suggests that mindfulness can help in the regulation of stress reactions [56,64,65]. Findings from a study conducted by Hülsheger et al. found that mindfulness was negatively associated with emotional exhaustion and positively associated with job satisfaction in a sample of 219 working adults [56]. Roeser et al. used 113 schoolteachers from Canada and the United States and demonstrated that mindfulness may lower levels of occupational stress and burnout [64]. Similarly, a study of 381 adults with diverse educational qualifications showed that the different facets of mindfulness were predictive of components of burnout [65]. These studies suggest that trait mindfulness can act as a personal resource which reduces burnout and work stress [56,65,66,67]. In fact, several studies have applied the JD-R model and tested for the moderating effect of mindfulness in relations between JD-R and various outcomes, such as burnout [66,68], health [69], work engagement [70], job satisfaction [71] and well-being [72]. In essence, mindfulness has been found to be a protective factor against negative work and health outcomes. Yet, a smaller body of evidence has begun to show that mindfulness may have a dual mediator/moderator role in the relation between stressors and health outcomes [73,74]. Taken together, the literature on mindfulness, particularly in the context of the JD-R model, has already shown the moderating effect of mindfulness. On the other hand, studies have yet to examine state mindfulness in a mediating role between JD-R and burnout among social workers. Thus, this study seeks to examine the role that state mindfulness may play in the relation between JD-R and burnout in a sample of Chinese social workers. 

### 2.5. Conceptual Framework

**Figure 1:**
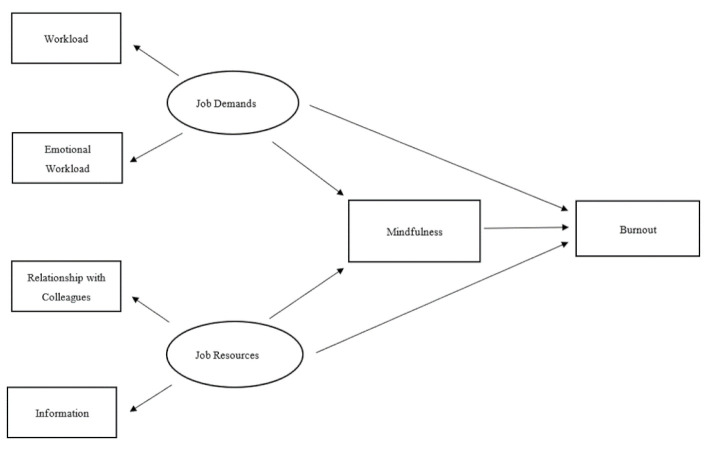
Conceptual model of JD-R, mindfulness, and burnout.

Based on JD-R theory and mindfulness frameworks [39,40,41,42,55,73], a conceptual model involving JD-R, state mindfulness, and burnout is proposed, as shown in Figure 1, to examine the mediational pathway between JD-R and burnout. This conceptual model posits that stressors such as JD may affect an individual’s moment-to-moment, or state, mindfulness by impairing their ability to stay aware of the immediate environment and/or to stay non-judgmental. Low state mindfulness may lead to emotional exhaustion and depersonalization, two key aspects of burnout. By contrast, resources such as JR may increase state mindfulness by improving ability to stay aware of the work environment and to remain non-judgmental. High state mindfulness can, in turn, lead to lower emotional exhaustion and depersonalization. Each of the latent exogenous factors, JD and JR, were operationalized with two exogenous observed variables. The manifest indicators of JD were workload and emotional workload, while the indicators of JR were relationship with colleagues and information. The structural model posits that burnout, an observed endogenous variable, and state mindfulness, an observed mediator, are affected by JD and JR. We hypothesize that:

1. Workload and emotional workload determine JD.
2. Relationship with colleagues and information determine JR.
3. JD reduce state mindfulness.
4. JR increase state mindfulness.
5. State mindfulness, along with JD and JR, have effects on burnout. Specifically, mindfulness and JR reduce burnout, while JD increase burnout.

## 3. Methods

### 3.1. Data and Sample

The data for the present study came from an anonymous online survey administered to social workers in Chengdu, China. Chengdu is a city that has experienced rapid development in social work [75,76]. Out of Chengdu’s 22 districts, we randomly selected two districts and, with the help of each district’s Civil Affairs Bureaus (CAB), contacted their respective social workers associations and agencies to recruit participants. Local CAB’s have records of all social workers and their associations and agencies. In Chengdu, all front-line social workers are required to be registered with the CAB in the district of their employment. In addition, all registered social workers who provide services to clients from other districts need to be on the record of those districts’ corresponding CAB. We obtained the list of social workers’ associations and agencies from the two CAB’s. Each district had around 600 social workers, who were employed by over 160 social work associations and agencies. These included: community social work centers; community development centers; community social services associations; home care service centers; elderly care centers; adolescent development centers; family development centers; and environmental awareness centers. The scope of practice among these social workers was varied. They provided services to various age groups (children and adolescent, adult, and geriatric populations) and all genders in different settings (community, elderly centers, school, and hospitals). Their work encompassed both direct practice and policy work.

The invitation to participate in the survey was sent to the respective social workers associations and agencies on 29 May 2021, with the endorsement of the local CAB. The invitations asked the associations and agencies to have social workers who worked in these two districts participate in the survey. Reminders to complete the survey were sent one week and two weeks after the initial invitation. Inclusion criteria for the study sample were all social workers who provided services in the sampled districts in Chengdu. Between the initial invitation and 29 May 2021, 915 social workers had responded to the surveys. We excluded 18 surveys from the final analysis due to incomplete answers. The final analytic sample contained data from 897 social workers, indicating a response rate of 75%. The research protocol was approved by the research review committee at the university of one of the coauthors in China. An informed consent process was implemented prior to the survey; individuals were informed that their participation was voluntary and that they could choose to stop the survey at any time. Participants were compensated with 5 RMB (1 USD).

### 3.2. Measures

The outcome variable, burnout, was measured using the Maslach Burnout Inventory, Human Services Survey (MBI-HSS) [77]. MBI-HSS is a self-scored survey with 22 items that measure three dimensions of burnout: emotional exhaustion, depersonalization, and personal accomplishment. The first dimension, emotional exhaustion, is the feeling of being “overextended” or exhausted by work. This refers to the psychological cost of JD. The second dimension, depersonalization, is defined by impersonal responses or feelings regarding clients or customers. The third and final dimension, personal accomplishment, refers to feelings of self-competence and achievement in work. The subscales represent a multidimensional concept of burnout. The psychometric soundness, reliability, and validity of the MBI-HSS have been verified in samples of professionals from various occupations, in different languages, and in different countries [78,79,80]. The internal reliability of the depersonalization scale, however, was between 0.65 and 0.70 in some samples [80,81]. Importantly, this study utilizes the Chinese version of MBI-HSS, which differs from the English version in that it contains 17 items rather than 22. Zhang et al. [82] administered the MBI-HSS to over 4800 Chinese police officers and found that 5 items had high factor loadings in multiple dimensions. These items were removed, leading to the 17-item Chinese version of MBI-HSS. This version of the MBI-HSS has shown good reliability (Cronbach’s alpha 0.71). The subscales similarly have good reliability: for emotional exhaustion (7 items), depersonalization (3 items), and personal accomplishment (7 items), the Cronbach’s alpha coefficients were 0.90, 0.75, and 0.78, respectively [82]. Responses to items in MBI-HSS range from 0 (never) to 6 (every day). We reversed the item scores in the personal accomplishment subscale so that higher scores indicated greater burnout. The total of all scores provided ranged from 0 to 102. The Cronbach’s alpha for all 17 items was 0.88 in this study. Meanwhile, the Cronbach’s alpha scores were 0.92, 0.87, and 0.88 for the emotional exhaustion, depersonalization, and personal accomplishment subscales, respectively. 

JD and JR were measured via Questionnaire sur les Ressources et Contraintes Professionnelles (QRCP) [42]. While QRCP is a multidimensional scale, we used two dimensions of JD (workload and emotional load) and two dimensions of JR (relationship with colleagues and information). These dimensions were selected based on the roles that social workers in China hold. The first JD dimension, workload, measures the degree to which respondents feel that they have too much work to do in the time that they have available. The second, emotional load, refers to those emotional JD that require individuals to cope with job-inherent emotions (e.g., frustration towards clients) [83] and/or organizationally desired emotions (e.g., remaining calm) [84,85]. The first dimension of JR, relationship with colleagues, measures team atmosphere and perceived potential to receive social support from coworkers. Finally, the information dimension measures the extent to which employees have access to job performance feedback. The dimensions—which contain 4 items each—had high Cronbach’s alpha values in Lequerre et al.’s study [42]. All had Cronbach’s alpha coefficients greater than 0.80 (workload, 0.84; emotional load, 0.83; relationship with colleagues, 0.87; information, 0.86). Item responses were scored on a 7-point Likert scale ranging from 1 (never) to 7 (always). Higher scores indicated higher levels of JD or JR. Each dimension’s total score ranged from 4 to 28. The Cronbach’s alpha was 0.87 for all 16 items in this study. The corresponding Cronbach’s alpha values for workload, emotional workload, relationship with colleagues, and information were 0.80, 0.68, 0.87, and 0.89, respectively.

We used the Five Facet Mindfulness Questionnaire (FFMQ) [52] to measure state mindfulness in the last two weeks. The FFMQ is a 39-item scale based on previous mindfulness scales, including the Mindful Attention Awareness Scale (MAAS) [86], the Kentucky Inventory of Mindfulness Skills (KIMS) [87], the Cognitive and Affective Mindfulness Scale (CAMS) [88], the Mindfulness Questionnaire (MQ) [89], and the Freiburg Mindfulness Inventory (FMI) [90]. The FFMQ measures mindfulness as a construct with five different facets: non-reactivity to inner experience; observing; acting with awareness; describing; and non-judging of experience. Nonreactivity to inner experiences reflects an individual’s ability to remain calm and objective when faced with thoughts or feelings that may usually elicit emotional responses. Observing measures an individual’s tendency to be aware of and recognize their thoughts and feelings. Acting with awareness indicates an individual’s ability to stay present in and aware of the moment while ignoring or sidestepping potential distractions. *Describing* refers to an individual’s capacity to recognize and label the thoughts and feelings that they experience. Nonjudging of experience involves the tendency towards objective consideration of thoughts and feelings and the rejection of assigning value to these thoughts and feelings. The psychometric properties of FFMQ have been well examined, showing high internal consistency and convergent and discriminant relationships with other variables in various populations across countries [52,91,92,93]. For example, each subscale had a high (greater than 0.75) Cronbach’s alpha coefficient (nonreactivity, 0.75; observing, 0.83; acting with awareness, 0.87; describing, 0.91; nonjudging, 0.87) [52]. Researchers have developed a short form of the FFMQ, with high internal consistency and validity [94,95]. Meng and colleagues developed a 20-item short form FFMQ, with four items per facet. They found that the psychometric properties of FFMQ were acceptable. The Cronbach’s alpha was 0.73 for their scale. Subscale Cronbach’s alpha coefficients were: nonreactivity, 0.66; observing, 0.75; acting with awareness, 0.80; describing, 0.79; nonjudging, 0.67 [95]. We used the Chinese version of the short form FFMQ and asked respondents to respond to the items according to the last two weeks. All items were rated on a 5-point Likert scale ranging from 1 (never) to 5 (always). Negative items were reversed coded so that higher scores in each item indicated higher levels of mindfulness. The total of all scores ranged from 20 to 100 for the scale, and 4 to 20 for each facet. The Cronbach’s alpha was 0.91 for the scale in this study, and the subscale Cronbach’s alpha coefficients were 0.77, 0.79, 0.81, 0.85, and 0.80 for nonreactivity, observing, acting with awareness, describing, nonjudging, respectively. 

### 3.3. Analytical Strategy

Descriptive analysis and Pearson’s correlation analysis were first undertaken to observe the sample characteristics and correlations among all variables. Then, we conducted structural equation modeling (SEM) analysis to examine the relations among JD-R, mindfulness, and burnout, while controlling for gender, age, and education. All controlled variables were assumed to have effects on mindfulness and burnout. SEM, unlike regression techniques, allows for the simultaneous examination of direct and indirect effects through mediating variables [96]. STATA software 16.0 was used for all analyses. In results that area not shown, we conducted regression analyses with extensive covariates, including personal and family characteristics, the results of which indicated that the relations among JD-R, mindfulness, and burnout were similar to those reported here.

## 4. Results

Table 1 presents the descriptive statistics of and correlations among the variables. The sample average burnout score was 53.9, while the sample average score of state mindfulness was 61.9. The sample reported high JD and JR; both averaged around 20 on scales that ranged from 4 to 28, suggesting that the social workers experienced high JD but also had support from coworkers and could access information from their employers. About 80% of the sample was female. The average age of the sample was 31.8, and around 60% of them had at least a college degree.

The results of correlation analyses were consistent with our hypotheses. The results indicated that burnout was negatively associated with state mindfulness (r = −0.40, *p* < 0.001) and JR (colleagues, r = −0.36, *p* < 0.001; information, r = −0.31, *p* < 0.001). Burnout was positively associated with JD (workload, r = 0.13, *p* < 0.001; emotional workload, r = 0.19, *p* < 0.001). State mindfulness had significant and positive correlations with JR (colleagues, r = 0.31, *p* < 0.001; information, r = 0.29, *p* < 0.001), age (r = 0.11, *p* < 0.001), and college education (r = 0.10, *p* < 0.01). Surprisingly, the JD dimension workload positively correlated with state mindfulness (r = 0.07, *p* < 0.05). JD and JR items were highly and positively correlated with each other. JD were also highly correlated with college education. Further, regression analysis suggested that the positive correlation between mindfulness and JD was driven by college education. 

**Figure 2:**
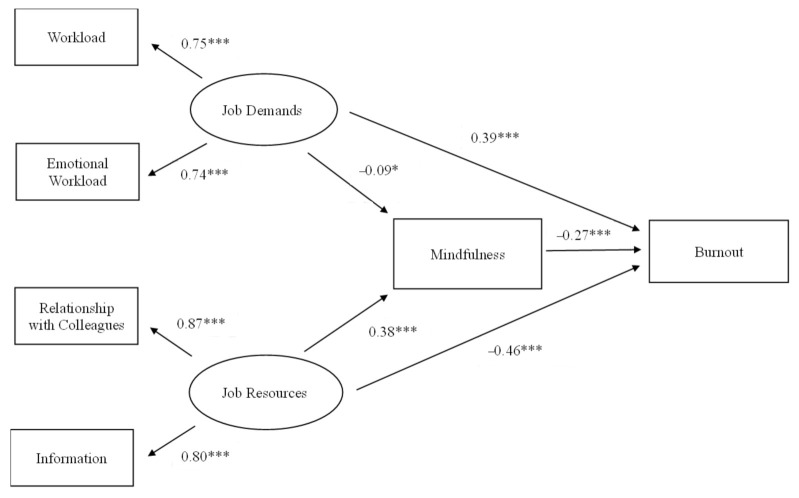
Standardized estimates of JD-R, mindfulness, and burnout model. * *p* < 0.05, *** *p* < 0.001.

The results of SEM analysis showed that the proposed model fit adequately to the data: χ^2^ (11) = 15.78, *p* > 0.05, CFI = 0.99, RMSEA = 0.02, SRMR = 0.01, Tucker–Lewis index = 0.99, and comparative fit index = 0.99. Figure 2 presents the standardized coefficients of the model. Full results of mediation analysis are listed in the Appendix A (see Table A1). All working conditions had significant loadings and were in the expected direction with the hypothesized JD and JR latent factors. JR were positively associated with state mindfulness (β =0.38, *p* < 0.001), while JD were negatively associated with state mindfulness (β = −0.09, *p* < 0.05). In addition, both JR (β = −0.46, *p* < 0.001) and state mindfulness (β =−0.27, *p* < 0.001) significantly reduced burnout. JD. On the other hand, JD significantly increased burnout (β = 0.39, *p* < 0.001). The above findings were consistent with our hypotheses and suggest that state mindfulness partially mediated the association between JD and burnout and between JR and burnout. The total effect of JR on burnout was −0.56. The indirect effect of JR via mindfulness was −0.10 (*p* < 0.001). The proportion of the effect mediated by state mindfulness was 0.18 (−0.10/−0.56). The total effect of JD on burnout was 0.42, and the indirect effect of JD through state mindfulness was 0.03 (*p* < 0.05). The proportion of JD’s effect on burnout that was mediated by state mindfulness was 0.07 (0.03/0.42). 

## 5. Discussion

Empirical evidence from studies using primarily Western samples has shown that JD-R have profound consequences on burnout and well-being [40,41,42]. Research also shows that mindfulness can improve emotion and self-regulation so that individuals can be more capable of recognizing, managing, and resolving emotions and life problems, as well as reducing burnout [56,65,66]. Less is known about whether these relations are the same in non-Western populations; further, little is known about the mediation effect of mindfulness in the relation between JD-R and burnout [26]. The present study used the JD-R model to examine how different categories of working conditions—JD and JR—are related to burnout and whether these relations are mediated by state mindfulness in Chinese social workers, an emerging profession with a high turnover rate. 

The descriptive statistics indicated that social workers must cope with high workload and emotional workload on the job. At the same time, the social workers in our sample reported high support from colleagues and availability of information from their employer, including performance feedback. The results of the correlation analysis showed that workload and emotional workload were strong predictors of JD, while relationship with colleagues and information were strong predictors of JR for social workers in China.

The SEM results provided support for the hypothesized dual process of burnout in Chinese social workers. The first process can best be described as an energy depletion process, starting with high job demands, which led to reduced state mindfulness and, in turn, burnout. The second process is motivational in nature and starts with JR. Social workers who could draw upon JR, such as support from colleagues and performance feedback, were more likely to have greater state mindfulness and, subsequently, less burnout. The magnitude of the estimates show that JR have greater effects on state mindfulness and burnout than JD. Indeed, the direct effect of JR on state mindfulness was strong (β = 0.38), and its total effect on burnout was high (β = −0.56). The relative numbers for JD were −0.09 and 0.42, respectively.

Taken together, these findings support and expand upon previous findings with the JD–R model among other occupational groups, showing that JD-R are important predictors of burnout in social workers specifically [30,31,32,33,39,41]. Thus, the underlying processes of energy depletion and motivation do not seem to differ between social workers and employees in other professions. Yet, the specific JD and JR may differ to some extent; in the present study, we found that relationships with colleagues and information were particularly relevant for social workers in China.

Our findings provide guidance for policy, practice, and research. First, given that the social workers in our sample reported high job and emotional workloads, and given the significant estimates of JD on mindfulness and burnout, agencies and organizations that employ social workers in China can adjust work conditions to prevent burnout in their employees. This includes reducing workload and providing support to make emotional workloads more manageable. For example, at present, few cities in China have adopted the practice of social work supervision [97], though supervision has been described as a crucial facilitator of safe and positive social work practice [98]. Supervision can allow social workers to reflect and explore emotional challenges on the job [98], acting as a JR that reduces the cost of emotional workload. 

In addition, the strong effects of JR on mindfulness and burnout suggest that employers can continue to maintain and support camaraderie among their staff. Making performance feedback accessible to social workers can also increase mindfulness and reduce burnout. Although these suggestions would require individual agencies and organizations to make changes that may reduce social worker burnout, uneven development across China [18,19,20,21,22] suggests that agencies and organizations in different areas of China may not be able to reduce JD or improve JR due to resource constraints. While findings of descriptive analysis suggested that the social workers had high JR at their disposal, this may not be the case for social workers from rural regions of China. As such, local, provincial, and government policies should ensure that sufficient funds and resources are allocated to agencies and organizations that employ social workers, perhaps with emphasis on those located in remote areas of China. With enough resources, employers may be able to better support their employees’ well-being.

Importantly, this study also indicates that mindfulness can be a critical point of intervention to prevent burnout among social workers in China, particularly those who live in Chengdu and cities like it. Thus, further studies are needed to assess whether mindfulness interventions may be adapted and administered to social workers to mitigate the effects of JD on burnout and add to the effects of JR on burnout. Past research has provided support for mindfulness-based interventions’ positive effects on mental health. Mindfulness-based stress reduction (MBSR), mindfulness-based cognitive therapy (MBCT), and mindfulness-based interventions (MBI) have all been shown to reduce psychological distress and promote well-being [99,100,101,102,103,104,105] across several age groups [102,103,104,105], highlighting the potential of implementing such interventions in the Chinese social worker population. At the same time, however, mindfulness did not fully mediate the relation between JD-R and burnout, indicating that MBIs are unlikely to eradicate burnout in social workers. As previously mentioned, organizational reform and the improvement of labor conditions are also important to protecting social workers from burnout.

While these results provide support for a partial mediational pathway between JD-R and burnout via mindfulness, further research is needed to investigate how these relations may differ according to different measurements and operationalization. This is important considering that JD-R, burnout, and mindfulness are all multi-dimensional constructs. For example, according to Demerouti [105], JD has five dimensions (i.e., physical workload, time pressure, recipient contact, physical environment, and shift work). JR, on the other hand, in Demerouti’s conceptualization, has six dimensions (i.e., feedback, rewards, job control, participation, job security, and supervisor support) [105]. Meanwhile, Lequeurre et al. [42] identified seven JD dimensions and seven JR dimensions. Due to the resource constraints of this study, we only focused on two JD and two JR dimensions that have previously been found to have significant effects on burnout [42]. Future studies can thus examine the extent to which other dimensions of JD and JR may affect mindfulness and burnout. Similar to JD-R, mindfulness has also been found to have several dimensions. Examples include non-reactivity to inner experience; observing; acting with awareness; describing; and non-judging of experience. Meanwhile burnout, as discussed earlier in this paper, is measured via three subscales, each representing its own dimension. Given the multi-dimensionality of our main variables, it is possible that the different dimensions of JD-R and mindfulness will differentially affect the different dimensions of burnout, and those who seek to use mindfulness interventions to protect social workers from burnout must consider the specific facets of mindfulness targeted by each.

The results of this study must be considered within the context of several limitations. First, the use of a cross-sectional dataset only allows us to approximate associative relations. To better evaluate causal relations, future research should utilize a longitudinal design to examine temporal order of JD-R, mindfulness, and burnout. Second, there are unobserved variables (e.g., job, personality, etc.) that could affect JD-R, mindfulness, and burnout, but these were not included in the study. Third, the data collected for this study were based on respondents’ self-reports. Our data are therefore subject to reporting errors, such as social desirability bias. To account for this, future studies may utilize triangulation of data by including reports from coworkers, family, and employers. In addition, this study was conducted during the global COVID-19 pandemic. While positive COVID-19 cases remained low in Chengdu at the time of survey (cumulative cases numbered under 170 in a city of over 16 million on 1 September 2021 [106]), the extent of how the pandemic affected JD-R, mindfulness, and burnout of social workers is unknown, warranting further study. Finally, this study analyzed data that were collected from social workers in Chengdu, China. While the sample size and high response rate increase our confidence, these findings may not be generalizable to the entire social worker population in China. Thus, further studies with social workers from geographically diverse areas of China are needed.

## 6. Conclusions

Applying the JD-R model of burnout, this study analyzed the relations among JD-R, mindfulness, and burnout in a sample of 897 social workers from Chengdu, China. Specifically, we investigated whether state mindfulness mediates the relations between JD-R and burnout. The findings were consistent with past cross-cultural research that found that JD are positively associated with burnout while JR are negatively associated with burnout. This study extends past research by providing evidence of these relations in a sample of Chinese social workers. Notably, the results of the SEM suggest that state mindfulness may serve as a potential mechanism that reduces JD’s effect on burnout and increases JR’s effect on burnout. Thus, mindfulness interventions may be useful to protect social workers from experiencing job burnout.

## Figures and Tables

**Table 1:** Descriptive Statistics and Correlations of Key Variables.

	Mean (S.D.)	1	2	3	4	5	6	7	8	9
1. Burnout (17–110)	53.9 (16.5)									
2. Mindfulness (41–88)	61.9 (6.4)	−0.40 ***								
3. JD-Workload (4–28)	20.0 (3.9)	0.13 ***	0.07 *							
4. JD-Emotional Workload (4–28)	18.5 (3.5)	0.19 ***	0.05	0.56 ***						
5. JR-Relationships (5–28)	20.9 (3.8)	−0.36 ***	0.31 ***	0.27 ***	0.21 ***					
6. JD-Information (4–28)	19.9 (3.8)	−0.31 ***	0.29 ***	0.27 ***	0.21 ***	0.70 ***				
7. Female (0–1)	0.8 (0.4)	−0.08 *	−0.03	−0.06	−0.13 ***	−0.04	−0.06			
8. Age (20–50)	31.8(7.3)	−0.16 ***	0.11 ***	0.03	0.01	0.05	0.07 *	−0.04		
9. Education-College or above (0–1)	0.6 (0.5)	0.12 ***	0.10 **	0.15 ***	0.17 ***	0.02	0.03	−0.05	−0.25 ***	

Note: N = 897. Numbers in parentheses show ranges of the variables. * *p* < 0.05, ** *p* < 0.01, *** *p* < 0.001.

## Data Availability

Data available on request due to privacy restrictions.

## Appendix A

**Table A1:** Full Results of Mediation Analysis.

Predictor	Dependent Variable	Direct Effect	Indirect Effect	Total Effect
JD	Mindfulness	−0.09 *	---	−0.09 *
JD	Burnout	0.39 ***	0.02 *	0.41 ***
JR	Mindfulness	0.38 ***	---	0.38 ***
JR	Burnout	−0.46 ***	−0.10 ***	−0.56 ***
Female	Mindfulness	−0.00	---	−0.00
Female	Burnout	−0.07 *	0.00	−0.07 *
Age	Mindfulness	0.12 ***	---	0.12 ***
Age	Burnout	−0.10 ***	−0.03 **	−0.13 ***
Education	Mindfulness	0.14 ***	---	0.14 ***
Education	Burnout	0.04	−0.04 ***	0.00
Mindfulness	Burnout	−0.27 ***	---	−0.27 ***

* *p* < 0.05, ** *p* < 0.01, *** *p* < 0.001.
